# Mechanism of action of 4‐substituted phenols to induce vitiligo and antimelanoma immunity

**DOI:** 10.1111/pcmr.12774

**Published:** 2019-03-18

**Authors:** Arthur Kammeyer, Karin J. Willemsen, Wouter Ouwerkerk, Walbert J. Bakker, Danielle Ratsma, Sebas D. Pronk, Nico P. M. Smit, Rosalie M. Luiten

**Affiliations:** ^1^ Department of Dermatology and Netherlands Institute for Pigment Disorders, Amsterdam University Medical Centers, Amsterdam Infection & Immunity Institute, Cancer Center Amsterdam University of Amsterdam Amsterdam The Netherlands; ^2^ Department of Clinical Epidemiology, Biostatistics and Bioinformatics, Amsterdam University Medical Centers University of Amsterdam Amsterdam The Netherlands; ^3^ Department of Clinical Chemistry and Laboratory Medicine Leiden University Medical Center Leiden The Netherlands

**Keywords:** immunotherapy, leukoderma, melanocytes, melanoma, occupational vitiligo, phenols, tyrosinase

## Abstract

Monobenzone is a 4‐substituted phenol that can induce vitiligo and antimelanoma immunity. We investigated the influence of the chemical structure on the biological activity of a series of structurally related 4‐substituted phenols. All phenols inhibited cellular melanin synthesis, and eight of ten phenols inhibited tyrosinase activity, using the MBTH assay. These phenols also induced glutathione (GSH) depletion, indicative of quinone formation and protein thiol binding, which can increase the immunogenicity of melanosomal proteins. Specific T‐cell activation was found upon stimulation with phenol‐exposed pigmented cells, which also reacted with unexposed cells. In contrast, 4‐tertbutylphenol induced immune activation was not restricted to pigment cells, analogous to contact sensitization. We conclude that 4‐substituted phenols can induce specific T‐cell responses against melanocytes and melanoma cells, also acting at distant, unexposed body sites, and may confer a risk of chemical vitiligo. Conversely, these phenols may be applicable to induce specific antimelanoma immunity.

**Significance**

Skin‐bleaching phenols have mostly been studied for their biochemical interaction with melanin synthesis and toxicity against melanocytes. This study links the biochemical characteristics of 4‐substituted phenols to their immunizing potential against pigmented cells, which may trigger vitiligo. This study thereby reveals the similarities and differences in mechanism of action of both known skin‐bleaching phenols, such as 4‐methoxyphenol, and other structurally related phenols that have not yet been associated with leukoderma or vitiligo. This study shows a broad range of phenols that may confer a risk of skin‐bleaching and chemical vitiligo. In addition, as previously shown for monobenzone, the immunizing potential of these phenols may be applicable to raise immune responses against melanoma.

## 1 INTRODUCTION

Many phenolic compounds are held responsible for skin bleaching or leukoderma (Bleehen, Pathak, Hori, & Fitzpatrick, 1968; Fisher, 2001). The depigmenting effect of these compounds, in particular 4‐substituted phenols, has been ascribed to direct toxicity to melanocytes (Manga, Sheyn, Yang, Sarangarajan, & Boissy, 2006; Manini, Napolitano, Westerhof, Riley, & d'Ischia, 2009; Naish, Holden, Cooksey, & Riley, 1988; Smit et al., 1992). Skin contact with phenols or catechols, such as monobenzyl ether of hydroquinone (MBEH or monobenzone, in this study referred to as 4‐benzyloxyphenol, BOP), can induce local depigmentation that can also spread to distant, unexposed body sites (occupational vitiligo). This depigmentation is clinically and histologically indistinguishable from vitiligo (Boissy & Manga, 2004; Vrijman et al., 2013). Systemic spread of the depigmentation indicates the presence of systemic reactivity against melanocytes. We have previously demonstrated the mechanism of action by which monobenzone induces immunity against melanocytes.(van den Boorn, Melief, & Luiten, 2011; van den Boorn, Picavet et al., 2011). Upon interaction with tyrosinase, monobenzone is converted into a reactive quinone that binds to thiol groups in tyrosinase or other melanosomal proteins (hapten formation), which increases their immunogenicity. Monobenzone also induces oxidative stress and the release of exosomes containing melanosomal proteins, which are taken up by dendritic cells, leading to their activation. These dendritic cells induce a specific immune response against melanocytes, resulting in vitiligo. The induction of melanocyte‐reactive immunity by monobenzone can be further enhanced in combination with immune‐stimulating agents, as a powerful depigmentation therapy (Webb et al., 2014).

Other skin‐bleaching phenols, like 4‐methoxyphenol (4‐hydroxyanisole), have extensively been studied for their interaction with tyrosinase, resulting in the inactivation of tyrosinase enzymatic activity and reactive quinone formation (Cooksey, Jimbow, Land, & Riley, 1992; Garcia Canovas et al., 1987; Naish, Cooksey, & Riley, 1988; Naish, Holden et al., 1988; Smit et al., 1992). The role of quinone formation in the skin depigmentation has been shown in animal models, in which quinone metabolites of phenols or catechols induced more extensive depigmentation than the parental compound (Tayama & Takahama, 2002). Moreover, the extent of depigmentation by catechols in vivo was dependent on quinone formation by tyrosinase and covalent binding to proteins (Menter, Etemadi, Chapman, Hollins, & Willis, 1993). However, the immunological mechanism of these quinones to induce skin depigmentation has not been described, except for monobenzone. Monobenzone, 4‐methoxyphenol, 4‐tertbutylphenol, and hydroquinone are known depigmenting agents, but differ in type of 4‐substituted side group and in the presence or absence of an ether link. Moreover, the mechanism of action of 4‐tertbutylphenol in inducing melanin inhibition and melanocyte death has been shown to differ from monobenzone (Hariharan et al., 2010; Kroll et al., 2005; Manga et al., 2006; Yang, Sarangarajan, Le Poole, Medrano, & Boissy, 2000). Therefore, the potential risk of 4‐substituted phenols to induce occupational vitiligo cannot easily be estimated based on their chemical structure. In this study, we investigated a series of structurally related 4‐substituted phenols for their biochemical and cellular effects and immunizing ability against pigmented cells.

Depigmenting phenols break immunological tolerance to melanocyte differentiation self‐antigens in the pathogenesis of vitiligo. On the other hand, these compounds represent an attractive approach to induce immunity in melanoma patients against melanocyte differentiation antigens that are shared by melanoma cells. This study therefore also provides insight in the usefulness of 4‐substituted phenols as antimelanoma agents. We have shown that the immunity induced by monobenzone also reacts against melanoma cells (van den Boorn, Picavet et al., 2011). In combination with the immune‐stimulating agents imiquimod and/or CpG, this immunity can induce melanoma regression in murine models (van den Boorn et al., 2010) and in melanoma patients (Teulings et al., 2018). Other phenols may also be applicable for melanoma immunotherapy. The clinical activity of 4‐methoxyphenol has already been shown for depigmentation therapy of vitiligo universalis (Njoo, Vodegel, & Westerhof, 2000). Here, we investigated the immunizing ability of a series of 4‐substituted phenols, structurally related to monobenzone (in this study referred to as BOP (4‐benzyloxyphenol)), to determine their immunizing ability and potential as antimelanoma agents.

## 2 MATERIALS & METHODS

### 2.1 Phenolic compounds and reagents

All 4‐substituted phenols of this study, 2‐hydroxymethyl‐5‐hydroxy‐γ‐pyrone (kojic acid), reduced glutathione (GSH), L‐3,4‐dihydroxyphenylalanine (L‐DOPA), 3‐methyl‐benzothiazolinone (MBTH), and mushroom tyrosinase were supplied by Sigma‐Aldrich, (Zwijndrecht, NL). *Ortho*‐Phtaldialdehyde (OPA) was supplied by Invitrogen (Molecular Probes, Thermo Fisher Scientific, Bleiswijk, NL). All phenols, MBTH, L‐DOPA, and OPA were dissolved in argon‐purged DMSO and kept in the dark at room temperature. Mushroom tyrosinase was dissolved in argon‐purged sodium phosphate buffer 50 mM pH 7.1 and immediately frozen at −140°C in small aliquots.

### 2.2 L‐DOPA assay for tyrosinase enzymatic activity

This assay measures dopachrome formation from L‐DOPA (or tyrosine) by tyrosinase by its absorbance at 475 nm. Phenols were incubated at a concentration of 100 or 300 µM with 10 U/ml mushroom tyrosinase in 50 mM sodium phosphate buffer pH 7.1 (NaP_i_) (Merck, Schiphol‐Rijk, NL) for 60 min at room temperature (RT) in the dark. Subsequently, L‐DOPA was added at a concentration of 250 µM. An incubation without test phenol served as a positive control. The formation of dopachrome was monitored on fixed intervals during maximal 2 hr. Fixed wavelength measurements were performed in plastic 1‐cm cuvettes on a Jasco V‐560 double‐beam spectrophotometer (Jasco Corp. Jas.co Benelux BV, de Meern, NL). The area under the curve (AUC) of samples with (B) or without (A) phenolic compounds were compared. The inhibition of tyrosinase activity on the conversion of L‐DOPA to downstream melanin precursors was calculated as (A/B) × 100%.

### 2.3 MBTH assay for tyrosinase enzymatic activity

Tyrosinase activity was measured by the MBTH assay as described (Winder & Harris, 1991) with minor modifications. Phenols were incubated at concentrations of 8, 20, or 50 µM with 10 U/ml mushroom tyrosinase in the presence of 1,000 µM MBTH in NaP_i_ buffer for 30 min. Subsequently, L‐DOPA was added at a concentration of 100 µM. An incubation without phenol served as a positive control. The formation of the dopaquinone–MBTH complex was monitored spectrophotometrically at 500 nm on fixed intervals during maximal 2 hr and analyzed for the percentage tyrosinase inhibition, as described for the L‐DOPA assay.

### 2.4 The GSH depletion assay

Glutathione in the reduced form (GSH, 6.1 mg) was dissolved in 100 µl pure water. DMSO (9.9 ml) was added to obtain a final concentration of 2 mM. This solution was argon‐purged. Phenols were incubated at concentrations ranging from 20 to 80 µM with 10 U/mL mushroom tyrosinase and 40 µM GSH in 980 µl sodium phosphate buffer (50 mM pH 7.1) for 10 min at RT. Subsequently, 500 µl of OPA reagent buffer was added, consisting of 200 mM sodium pyrophosphate (NaPP), 5 mM disodium EDTA, and pH adjusted to 8.5 with 2 M HCl. This NaPP buffer was used at a favorable pH for the interaction of GSH with o‐phtaldialdehyde (OPA) to obtain a fluorescent reaction product, between pH 8 and 10. Finally, 20 µl of 30 mM OPA in DMSO was added (400 µM OPA final concentration) and the sample was allowed to react for 45 min. Fluorescence (340/450 nm) was measured in a 24‐well flat‐bottomed plate by a Fluo Star Optima apparatus (BMG LABTECH, GmbH, Ortenberg, Germany; Cohn & Lyle, 1966). The fluorescence level of non‐depleted GSH was set to 100%, and 1,2‐quinone‐induced GSH depletion was related to this value.

### 2.5 Cell lines and culture conditions

Human melanoma cell lines Mel 88.23 (SK‐MEL‐5), melWBO and Mel136.2, murine fibroblast L cells transfected with CD40L and human keratinocyte cell line HaCaT were cultured in IMDM supplemented with 8% FCS, 2 mM glutamine, 50 U/ml penicillin, and 50 mg/ml streptomycin (Gibco, Thermo Fisher Scientific, Bleiswijk, NL) at 37°C and 5% CO_2_. The murine melanoma cell line B16F10 was cultured in RPMI1640 (Gibco, Thermo Fisher Scientific, Bleiswijk, NL) with 8% FCS and supplements as above. The cells were passaged twice a week at a standard cell density 0.5 × 10^6^ cells in a T75 flask or 1.0 × 10^6^ cells in a T150 flask (Corning, New York, USA) by washing with phosphate‐buffered saline (PBS, Fresenius, Zeist, the Netherlands) and cell detachment with 2 mM EDTA (Sigma‐Aldrich). Adherent HaCat cells were passaged in culture by 0.05% trypsin/EDTA detachment (Gibco, Thermo Fischer Scientific).

### 2.6 Cell viability assay

Human melanoma cell lines Mel88.23, Mel136.6 and/or MelWBO, and HaCat cells were seeded at a density of 8,000 cells per well in 96‐well flat‐bottom well plate (Corning, New York, USA) and cultured overnight. Phenolic compounds were added at 1,000 µM and a twofold dilution range to 7.8 µM and cultured for 72 hr at 37°C and 5% CO_2_. Control incubation was performed without phenol. After incubation, cells were washed twice with PBS and fresh medium was added, to remove phenol and avoid their potential reaction with (3‐(4,5‐dimethylthiazol‐2‐yl)‐2,5‐diphenyltetrazolium bromide (MTT). Cell viability was measured by the CellTiter96 non‐radioactive cell proliferation assay (Promega, Madison, MA) containing MTT, according to the manufacturer's protocol. Optical density was measured at 570 nm (Versamax ELISA microplate reader, Molecular Devices, Sunnyvale, CA) with 650 nm as reference wavelength to reduce the background. The percentage of cytotoxicity was calculated relative to the viability of untreated cells. The inhibitory concentration (IC)50, IC25, and IC12.5 were determined as the concentration showing a 50%, 25%, or 12.5% decrease in cell viability, respectively.

### 2.7 Tyrosinase protein expression

Mel 88.23 cells were seeded at 0.5 × 10^6^ cells/well in a 6‐well plate and the next day incubated with one of the nine phenols at IC25 concentration (Table 1). After 72 hr, cells were harvested and lysed as described before (de Bruin et al.., 2016). The protein concentration was determined using the Bradford assay (#39222, Serva, Heidelberg, Germany). Equal protein amounts were analyzed by Western blot, following SDS–PAGE (7.5% gel) and transfer as described before (van den Boorn, Picavet et al., 2011), using tyrosinase‐specific antibody T311 (#35‐6000 Invitrogen, Sanbio, Uden, NL) and anti‐actin antibody SC‐1616 (Santa Cruz Biotechnology, Heidelberg, Germany).

**Table 1:** Toxicity and immunising capacity of 4‐substituted phenols

Compound	Abbrev.	Inhib. Conc^a^	Conc^b^	CD8 T‐cell activation^c^				CD4 T‐cell activation^c^			
				CD137	TNFα	INFγ	ORR^d^	CD137	TNFα	INFγ	ORR^d^
4‐Benzyloxyphenol^e^	BOP^f^	IC25	10 µM	42.9	57.1	28.6	43	42.9	42.9	42.9	43
		IC12.5	5 µM	12.5	62.5	50	63	12.5	12.5	37.5	50
4‐Phenoxyphenol	POP	IC25	25 µM	25	50	12.5	50	25	37.5	12.5	50
		IC12.5	12.5 µM	25	75	12.5	75	25	37.5	12.5	63
4‐Methoxyphenol	MOP	IC25	50 µM	0	50	0	50	12.5	25	25	38
		IC12.5	35 µM	25	62.5	12.5	63	37.5	37.5	37.5	63
4‐(n‐Hexyl)oxyphenol	HOP	IC25	4 µM	12.5	50	12.5	63	12.5	25	12.5	25
		IC12.5	2 µM	25	62.5	37.5	75	25	25	37.5	50
4‐Benzylphenol	BP	IC25	130 µM	12.5	37.5	37.5	50	12.5	25	25	63
		IC12.5	95 µM	25	37.5	12.5	50	25	12.5	50	50
4‐Phenylphenol	PhP	IC25	200 µM	50	62.5	0	75	50	37.5	0	75
		IC12.5	100 µM	12.5	50	0	50	25	25	25	63
4‐Methylphenol	MP	IC25	250 µM	12.5	62.5	12.5	63	25	37.5	37.5	63
		IC12.5	125 µM	12.5	50	25	50	12.5	37.5	37.5	63
4‐(n‐Pentyl)phenol	PP	IC25	100 µM	37.5	62.5	12.5	63	37.5	50	12.5	63
		IC12.5	50 µM	25	25	0	25	25	37.5	25	38
4‐(*tert*‐Butyl)phenol	TBP	IC25	200 µM	37.5	75	75	88	37.5	37.5	75	100
		IC12.5	100 µM	37.5	75	37.5	75	37.5	37.5	62.5	75

a Inhibitory concentration used in T‐cell activation assays.

b Inhibitory concentration as determined by the MTT toxicity assay.

c T‐cell activation as analyzed by CD137 expression and TNFα or IFNγ production. Data are expressed as response rate (%) of immunising activity in 8 donors.

d Overall response rate (ORR) of unique responders with at least 1 positive T‐cell activation marker (*n* = 8).

e Also known as monobenzone.

f *n* = 7 donors in T‐cell activation assays of BOP.

### 2.8 Determination of cellular melanin concentrations

Human melanoma cell line Mel 88.23 (SK‐MEL‐5) and murine melanoma cell line B16F10 were seeded at 0.5 × 10^6^ per well in 6‐well plate and cultured overnight in the presence of 1 mM L‐DOPA to stimulate melanin synthesis. Phenols were added to the cells at IC50 concentration and a twofold dilution range and cultured for 72 hr. Subsequently, cells were washed in PBS, 300 µl 1 M sodium hydroxide was added, and melanin was dissolved at 70°C for 120 min. Optical density of 200 µl of the dissolved cell solution, transferred to a 96‐well flat‐bottom microplate, was measured at 405 nm in a Versamax ELISA microplate reader. The melanin content of samples was calculated by a calibration curve of synthetic melanin (Sigma‐Aldrich) ranging from 0.49 to 500 µM melanin. The percentage inhibition of melanin synthesis in melanoma cells by phenol was calculated relative to untreated cells. Non‐pigmented cell lines HaCat and L cells served as negative controls for human and murine melanoma cells, respectively. A Bradford protein assay (Bio‐Rad, Utrecht, NL) was performed to relate the melanin content to the protein concentration, using a calibration curve of bovine serum albumin (BSA) in 1 M NaOH.

### 2.9 T‐cell activation assays

PBMC were isolated by Ficoll density gradient centrifugation (LymphoPrep, PROGEN, Sanbio, Uden, NL) from anonymous healthy donor blood provided by Sanquin Blood Supply Foundation (Amsterdam, NL), according to the Declaration of Helsinki Principles. Immature monocyte‐derived dendritic cells (DC) were generated as described (de Jong et al., 2002). DC‐T cell autologous cocultures and restimulation were performed as described (van den Boorn, Picavet et al., 2011), with minor modifications as described below. Melanoma cell lines were pre‐incubated overnight with phenols at IC25 and IC12.5 concentrations or left unexposed and added to immature DC. Subsequently, autologous T cells were added and cultured for 7 days. At day 7, the cocultures were split into four parallel cultures and restimulated overnight with autologous DC pre‐incubated with either phenol‐exposed melanoma cells, phenol‐exposed HaCat cells, unexposed melanoma cells, or unexposed HaCat cells. T‐cell activation in the cocultures was analyzed by flow cytometry for T‐cell activation marker CD137 and the production of TNFα and IFNγ, as described (Tjin et al., 2011; van den Boorn, Picavet et al., 2011). Cells stained with FITC‐conjugated CD137‐specific Ab (clone 4B4‐1, eBioscience, Thermo Fisher Scientific, Bleiswijk, NL), APC/Fire750™‐conjugated anti‐CD8 Ab (clone SK1) and APC‐conjugated anti‐CD4 Ab (Clone SK3), both from Biolegend, ITK Diagnostics, Uithoorn, NL, followed by fixation and permeabilization in fixation buffer (Biolegend, ITK Diagnostics, Uithoorn, NL)) and intracellular staining permeabilization wash buffer (Biolegend, ITK Diagnostics, Uithoorn, NL) and staining with PerCP/Cy5.5‐conjugated TNFα Ab (clone MAb11), PE/Cy7‐conjugated IFNγ Ab (clone B27). Both antibodies were obtained from Biolegend (ITK Diagnostics, Uithoorn, NL). In addition, T‐cell activation during the coculture was measured by IFNγ ELISA of the supernatant at day 7 (eBioscience, Thermo Fisher Scientific, Bleiswijk, NL).

Data were analyzed as follows: Immunizing ability of 4‐substituted phenols was measured by overall response rate (ORR). ORR was defined as the percentage of unique responders with at least one positive T‐cell activation marker (CD137, TNFα, and IFNγ). Response was defined as an increase of two times standard error of the mean (*SEM*), calculated for all donors using the unexposed melanoma cell lines. We determined response for each T‐cell activation marker in both the CD8+ (cytotoxic) T cell and the CD4+ (helper) T cell population at IC25 and IC12.5 concentrations. All analyses were performed using R version 3.5.0.

## 3 RESULTS

### 3.1 4‐Substituted phenols show increased absorbance in the standard L‐DOPA assay for tyrosinase activity

**Figure 1:**
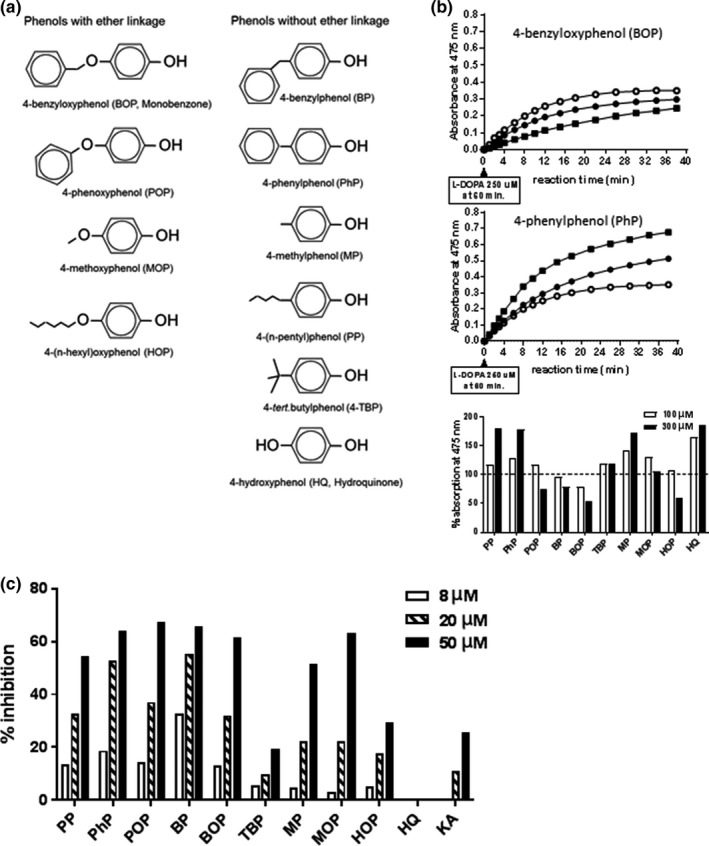
Tyrosinase inhibition by 4‐substituted phenols. (a) Chemical structure of the 4‐substituted phenols analyzed. (b) Tyrosinase inhibition assay with L‐DOPA in the absence (O) or presence of 100 µM BOP or PhP (●) or 300 µM BOP or PhP (■), showing increased absorbance of the reaction mixture at 475 nm in the presence of PhP and suggesting increased dopachrome formation and tyrosinase activity by PhP. *Lower panel*: Effect of 4‐substituted phenols on the absorbance at 475 nm in the tyrosinase L‐DOPA assay, quantified as area under the curve (AUC) and normalized to tyrosinase activity in the absence of phenol. (c) Inhibitory effect of 4‐substituted phenols on tyrosinase activity as measured in the MBTH assay

Previous data from our group and others have shown the skin‐bleaching and vitiligo‐inducing mode of action of monobenzone (van den Boorn, Picavet et al., 2011) and 4‐tertbutylphenol (TBP) (Hariharan et al., 2010; Kroll et al., 2005; Manga et al., 2006; Yang et al., 2000). Monobenzone or 4‐benzyloxyphenol (BOP) contains an ether‐linked benzyl group. It is, however, not known whether the structurally related phenol without ether linkage, 4‐benzylphenol, would have similar skin‐bleaching and vitiligo‐inducing ability. In this study, we investigated the biochemical, cellular, and immunological characteristics of nine structurally related 4‐substituted phenols (Figure 1a). We analyzed a series of commercially available 4‐substituted phenols with and without ether linkage (Figure 1a) for their inhibitory effect on tyrosinase activity, melanin synthesis, and their pigment cell‐specific immunizing ability. Hydroquinone and kojic acid were included as positive controls in the biochemical analyses.

We tested the phenols for their ability to inhibit tyrosinase activity using the classic mushroom tyrosinase activity assay based on the conversion of L‐DOPA into dopachrome and absorbance at 475 nm. Most of the phenols, including 4‐(n‐pentyl)phenol (PP), 4‐phenylphenol (PhP), 4‐phenoxyphenol (POP), TBP, 4‐methylphenol (MP), 4‐methoxyphenol (MOP), and control compound hydroquinone, showed an increase in absorbance at 475 nm, suggesting enhanced dopachrome formation and stimulation of tyrosinase activity, whereas others, including 4‐benzylphenol (BP), BOP, and 4‐(n‐hexyl)oxyphenol (HOP), showed a decrease in absorbance at 475 nm (Figure 1b). No increase in absorbance was seen in the absence of tyrosinase, indicating that there was no interaction of the phenols with DOPA. Figure 1b shows the gradual increase in absorbance in the presence of PhP and a decrease in the presence of BOP. The relative absorbance as measured by the area under the curve (AUC) of all tested phenols is shown in Figure 1c. These results did not correlate with the in vivo skin‐bleaching properties of MOP and HQ. Apparently, the quinone that was generated from the phenol by tyrosinase interfered with the detection of dopaquinone at 475 nm, thereby masking their inhibitory effect on tyrosinase activity.

### 3.2 The MBTH assay, an improved assay to determine inhibition of tyrosinase activity by 4‐substituted phenols

Due to the interference of the 4‐substituted phenols with the outcome of the standard L‐DOPA tyrosinase activity assay, we also used a different tyrosinase assay using 3‐methyl‐benzothiazolinone (MBTH) with improved specificity and sensitivity to measure tyrosinase activity in vitro (Winder & Harris, 1991). This method is based on MBTH, an agent that binds to a quinone and is detectable by its absorbance at 500 nm. In this MBTH assay, the formation of test phenol‐derived quinone did not interfere with the measurement of tyrosinase activity. Control incubations without tyrosinase remained at baseline level, indicating that there was no interaction with DOPA or MBTH. Moreover, the MBTH assay appeared to be up to tenfold more sensitive. The results of the MBTH assay also revealed the variation in tyrosinase inhibitory activity among phenols; BP and PhP inhibited tyrosinase activity more strongly than BOP, PP, and POP were comparable to BOP, while the others showed less inhibition than BOP (Figure 1c). The two strongest inhibitors, BP and PhP, belong to the group of non‐ether phenols, although this structural feature did not seem to be discriminative, since ether‐linked phenols POP and BOP were superior to PP. Well‐known skin‐bleaching compound kojic acid (KA) did not inhibit tyrosinase enzyme activity and HQ appeared to stimulate, rather than inhibit, tyrosinase activity, as was observed in the standard L‐DOPA tyrosinase activity assay.

### 3.3 Binding of 4‐substituted phenol‐derived quinones to protein thiol groups

**Figure 2:**
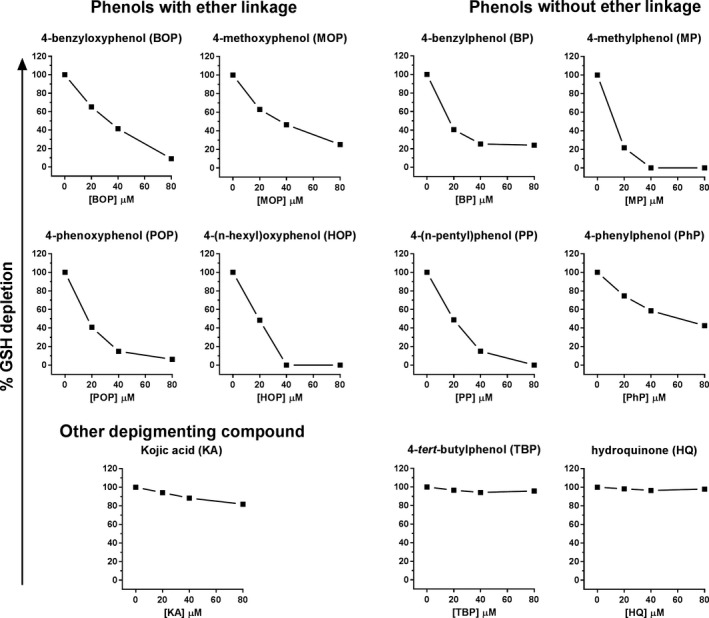
Reactive quinones bind free GSH, as indication of protein thiol binding. Binding of quinone to glutathione (GSH) resulting in depletion of free GSH was analyzed as surrogate marker for quinone formation and protein thiol binding. GSH depletion was measured by the decrease in fluorescence at 340/450 nm, relative to GSH levels in the absence of phenol (100%)

We next investigated whether the reaction of the 4‐substituted phenols with tyrosinase resulted in quinone formation that bind to thiol groups of proteins. This reaction can give rise to melanosomal protein‐hapten complexes with increased immunogenicity (Ewens, Wulferink, Goebel, & Gleichmann, 1999; Menter et al., 1993; Nazih, Benezra, & Lepoittevin, 1993), as we have previously shown for monobenzone (BOP) (van den Boorn, Picavet et al., 2011). We tested this in a GSH depletion assay that measures the binding of phenol‐derived quinone to glutathione (GSH), a tripeptide (Glu‐Cys‐Gly), as a model for proteins with thiol group. The reaction of phenol with tyrosinase was performed in the presence of GSH, during which GSH reacts with quinones forming a non‐fluorescent complex, while remaining free GSH forms a fluorescent complex upon addition of the OPA reagent. Phenol‐derived quinone binding to GSH was determined by the depletion of free GSH. The more quinone is formed from a phenol and tyrosinase, the more GSH will be depleted. GSH could also be depleted during the reaction by oxidation of GSH to GSSG, but this was of minor influence on the test outcome. Figure 2 shows the level of glutathione depletion by the 4‐substituted phenols at concentrations ranging from 0 to 80 µM. Eight out of ten phenols showed GSH depletion, indicating that the quinones derived from these phenols by tyrosinase bind thiol groups and can potentially give rise to protein‐hapten complexes. Most efficient quinone‐GSH binding was seen with HOP and MP, followed by POP and PP, whereas the effect of MOP and BP was comparable to BOP. PhP displayed less quinone‐GSH binding than BOP. In analogy to the tyrosinase inhibition data (Figure 1c), TBP and hydroquinone did not induce any GSH depletion, whereas kojic acid induced only low levels of GSH depletion (Figure 2).

### 3.4 Inhibition of melanin synthesis by 4‐substituted phenols

Besides the effects of tyrosinase activity, we further investigated the inhibitory effects of the phenols on the melanin synthesis by pigmented cells in culture. To this end, we first determined the toxicity of the phenols by incubating melanoma cells for 72 hr in the presence of a phenol, after which cell viability was analyzed. Table 1 shows the inhibitory concentration (IC)25 and IC12.5, representing the concentration at which 25 or 12.5% of cells have died during phenol incubation, respectively. The results show a large spread in toxicity of phenols. HOP is most toxic to pigmented cells, followed by BOP, POP, and MOP, whereas the other phenols were less toxic.

**Figure 3:**
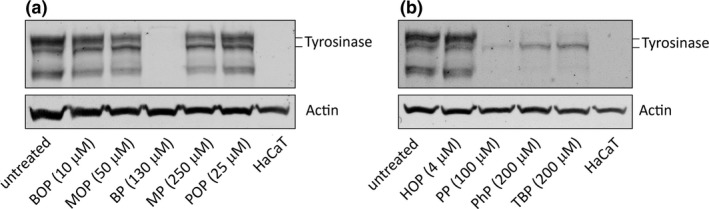
BP, PP, PhP, and TBP reduce tyrosinase protein levels, while the other phenols do not (a) Immunoblot showing tyrosinase protein levels (upper panel) in Mel 88.23 cells exposed for 72 hr to the indicated phenols (IC25 concentration) compared to untreated Mel 88.23 cells. Human keratinocytes (HaCaT cells) serve as negative control for tyrosinase expression. The two black bars indicate different tyrosinase forms (a mature 80‐kDa and an immature 70‐kDa form; Halaban, Cheng, Svedine, Aron, & Hebert, 2001). Actin immunostaining was performed as a loading control (lower panel). (b) Similar analyses as (a) for the effect of HOP, PP, PhP and TBP

Next, we analyzed the effect of the phenols on tyrosinase protein levels by Western blot. As shown in Figure 3, four phenols, BP, PP, PhP, and TBP, reduced tyrosinase protein levels in pigmented melanoma cells, whereas the other phenols did not affect tyrosinase levels. This confirms the previously reported inhibiting effect of TBP (Yang & Boissy, 1999) and our previous data of monobenzone (BOP) not affecting tyrosinase levels (van den Boorn, Picavet et al., 2011).

**Figure 4:**
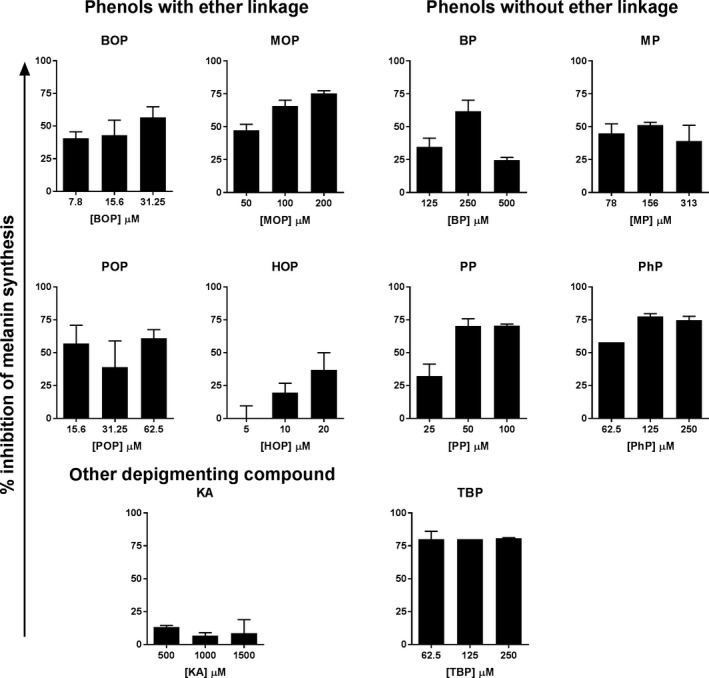
Inhibition of melanin synthesis by 4‐substituted phenols. Human and murine pigmented melanoma cell lines mel88.23 (SK‐MEL‐5) and B16.F10 were cultured in the presence of phenols at IC50 and a twofold dilution range for 72 hr. Figure shows the melanin inhibition in mel88.23 cells (baseline pigmentation: 18 ng melanin/µg protein, L‐DOPA induced pigmentation: 91 ng melanin/µg protein). Similar results were obtained in B16.F10 cells

Considering this variety of phenols on cellular toxicity and tyrosinase levels, we subsequently analyzed the effect of phenols on cellular melanin synthesis. For this, we incubated the cells with the phenols at concentrations with equal cell viability, that is the IC50 and a twofold dilution range. All nine phenols inhibited melanin synthesis in pigmented melanoma cells (Figure 4), whereas kojic acid only showed minimal inhibition. TBP most strongly inhibited melanin synthesis. MOP, PhP, and PP inhibited melanin synthesis more strongly than BOP, whereas POP and MP were comparable to BOP. The strong inhibitory effect on melanin synthesis by TBP, PhP, and PP likely results from the decreased tyrosinase protein expression and activity. HOP showed the least melanin inhibition. Inhibition of melanin synthesis by BOP, MOP, HOP, PhP, and PP was dose dependent, but not for BP, MP, POP, and TBP at the concentrations tested.

### 3.5 Immunizing ability of 4‐substituted phenols

We next investigated whether the phenols can induce immune activation against pigmented cells in healthy donors, as we have shown for monobenzone (BOP) (van den Boorn, Picavet et al., 2011). To this end, pigmented human melanoma cells were exposed to a phenol, loaded on dendritic cells as antigen‐presenting cells, and cultured with autologous T cells. After one week, activation of the T cells in the coculture was analyzed by flow cytometry. Phenol exposure of melanoma cells was performed at both IC25 and IC12.5, in order to have two levels of phenol‐induced melanoma cell apoptosis or necrosis in the activation assays. T‐cell activation was analyzed by the increased expression of the activation marker CD137 and by the production of cytokines TNFα and IFNγ, which are associated with a type 1 T‐cell response often observed in vitiligo. Activation of both CD8+ (cytotoxic) T cells and CD4+ (helper) T cell responses were analyzed in 8 healthy donors. The results are summarized in Table 1, showing the percentage of donors with T‐cell responses. Since some donors showed T‐cell activity for multiple markers, the overall response rate (ORR) was determined as the percentage of unique responders with at least one positive T‐cell activation marker. All phenols tested were able to induce CD8+ T cell activation in 25%–75% of donors at IC 12.5 and in 43 to 88% of donors at IC25. The phenols also induced CD4+ T cell activation in 38%–75% of donors at IC12.5 and in 25 to 100% of donors at IC25. These results indicate that exposure of pigmented cells to low doses of 4‐substituted phenols can already induce T‐cell activation.

T‐cell activation was not induced by all 4‐substituted phenols in all donors. The ORR data show that most phenols induced T‐cell responses in part of the donors. However, different donors reacted to a different subset of phenols, with overlap in reactivity between donors, and every donor reacted to one or more phenols. This suggests that these phenols all confer a potential risk of skin‐bleaching and vitiligo upon skin exposure.

**Figure 5:**
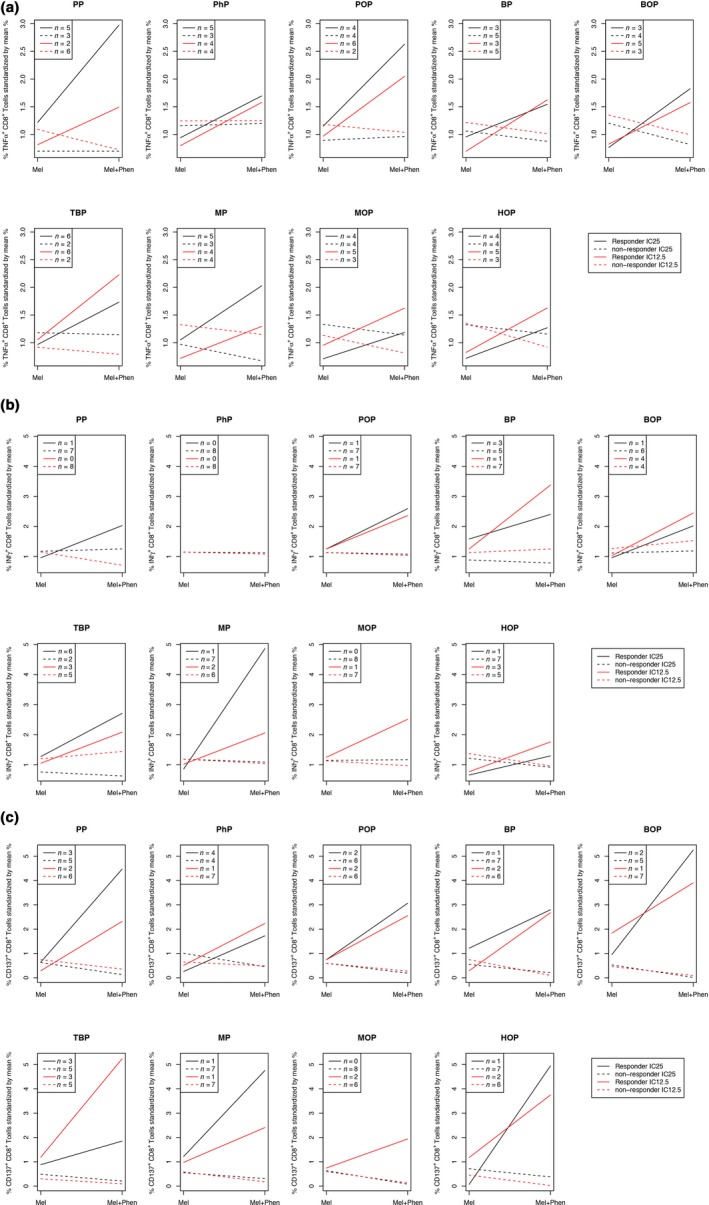
Induction of CD8+ T cell responses by 4‐substituted phenols. Human T cells were cultured with autologous dendritic cells loaded with phenol‐exposed (IC25 and IC12.5) or unexposed melanoma cells for 1 week and tested for their reactivity against phenol‐exposed melanoma cells and unexposed melanoma cells. Panels show the CD8+ T cell activation in responding donors and non‐responding donors against phenol‐exposed cells (Mel+Phen) at IC25 and IC12.5, as compared to unexposed cells (Mel), as measured by the production of TNFα (a), IFNγ (b), and CD137 expression (c). *n*, number of responding or non‐responding donors per test group

We further analyzed whether phenol exposure of pigmented cells leads to more T‐cell activation than unexposed pigmented cells (Figure 5 and Supporting Information Figure S1). T cells were stimulated for one week with DC loaded with phenol‐exposed melanoma cells or unexposed melanoma cells and subsequently tested for T‐cell activation. In responding donors, more T‐cell activation was seen upon stimulation with phenol‐exposed melanoma cells than unexposed melanoma cells. In non‐responding donors, T‐cell activation upon phenol‐exposed cell stimulation was comparable to, or lower than, without phenol exposure. Figure 4 shows the data of CD8 T cell responses as measured by the production of TNFα (Figure 5a), IFNγ (Figure 5b), or CD137 expression (Figure 5c). The CD4 T cell responses upon stimulation with phenol‐exposed or unexposed cells are shown in Supporting Information Figure S1. These results show that phenol‐exposed melanoma cells induce more T‐cell activation, than unexposed cells. This indicates that phenol exposure increases the immunogenicity of pigmented cells.

**Figure 6:**
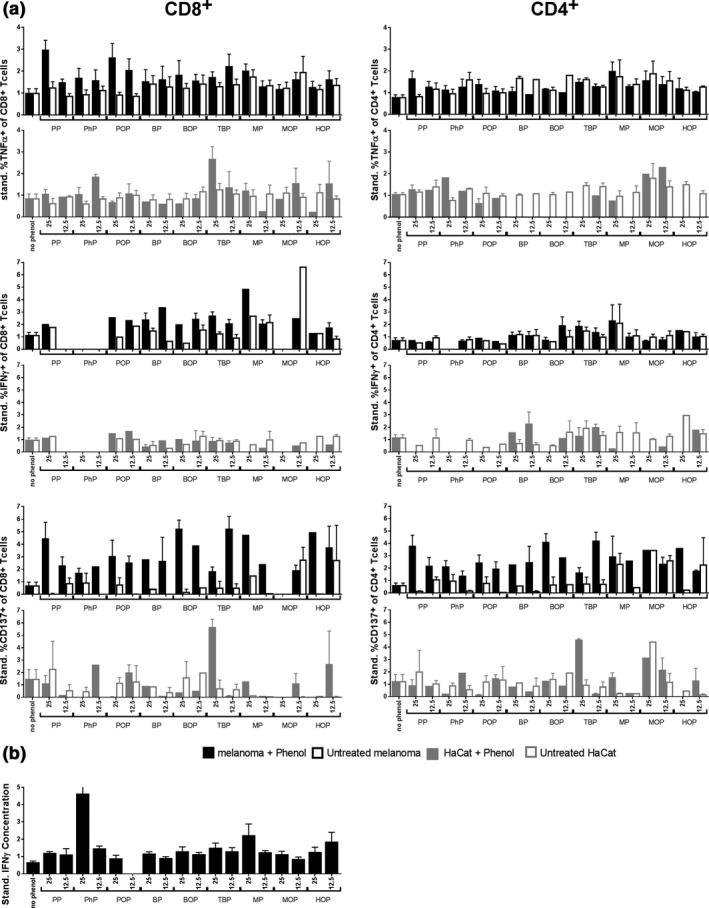
Pigment cell specificity of the phenol‐induced T‐cell activation. Human T cells were cultured with autologous dendritic cells loaded with phenol‐exposed (IC25 and IC12.5) or unexposed melanoma cells for 1 week and tested for their reactivity against phenol‐exposed or unexposed melanoma cells and against phenol‐exposed or unexposed keratinocytes (HaCat). (a) Panels show the percentage CD8+ and CD4+ T cell activation in responding donors (standardized by mean), as measured by the production of TNFα, IFNγ, and CD137 expression. (b) Total T‐cell activation against phenol‐exposed melanoma cells during one week culture, as measured by IFN γ ELISA

We next investigated whether the T‐cell responses induced by phenol‐exposed melanoma cells could also recognize unexposed melanoma cells. This would be indicative to what extent the phenol‐induced T‐cell response could induce depigmentation (and vitiligo) at distant unexposed body sites. To this end, T cells were first stimulated with phenol‐exposed melanoma cells and subsequently tested for the recognition of unexposed melanoma cells. Figure 6 shows the recognition of exposed and unexposed melanoma cells in responding donors by both CD8+ T cells and CD4+ T cells, as analyzed by T‐cell activation markers TNFα, IFNγ, and CD137. In general, the T cells recognized both exposed melanoma cells (black bars) and unexposed melanoma cells (white bars). Phenol‐exposed cells induced more CD137 expression by T cells than unexposed cells, but this difference was smaller for the cytokines TNFα and IFNγ. The results in Figure 6a display the level of T‐cell activation at day 7, as analyzed by flow cytometry. However, we noticed that the kinetics of the T‐cell response may vary between phenols. Figure 6b shows the accumulated levels of IFNγ produced during the 7‐day stimulation as analyzed by ELISA. Low levels of T‐cell activation by PhP were found by flow cytometry, but the ELISA of the 7 day culture supernatant revealed high IFNγ production, indicative of T‐cell activation before day 7. These results show that phenol exposure lowers the threshold for activation of (autoimmune) T‐cell responses against pigmented cells.

We further addressed the target specificity of the phenol‐induced T‐cell responses by investigating their recognition of other skin cells, in particular keratinocytes. This reveals to what extent phenol exposure induces T‐cell responses that only eliminate pigmented cells or also attack other skin cells resulting in more general skin damage. Phenol‐melanoma cell‐stimulated T cells recognized DC loaded with keratinocyte cell line HaCat to a lesser extent than DC loaded with melanoma cells (Figure 6, gray panels). Also phenol‐exposed HaCat cells were less recognized than phenol‐exposed melanoma cells. An exception was TBP, inducing T‐cell responses that recognized both phenol‐exposed melanoma cells and phenol‐(un)exposed Hacat cells, corresponding to the mode of action of contact sensitizers. Our data show for the first time the immunizing ability of the known skin‐bleaching compound MOP and suggest that BOP and MOP have a similar mechanism of action.

### 3.6 Combined analyses of biochemical, cellular, and immunizing effects of 4‐substituted phenols

In order to evaluate the influence of biochemical and cellular effects on the immunizing ability, the characteristics of the 4‐substituted phenols were compared those of BOP (Table 2). Since the melanocyte destruction in vitiligo is predominantly mediated by CD8+ T cell responses that are specific for pigmented cells, we combined the induction of CD8 T cell activation with the pigment cell specificity of the response, as primary read out for vitiligo‐inducing activity. Table 2 shows that the presence of an ether link in chemical structure of the compound does not determine its biochemical effects and immunizing ability, since these effects were found in both phenols with and without ether link. Similarly, the level of tyrosinase inhibition and cellular melanin synthesis inhibition did not show clear associations with immunizing ability. All phenols tested, except TBP and hydroquinone, induced (some level of) quinone binding and pigment cell‐specific T‐cell responses, but no clear association was found between the level of quinone binding and pigment cell‐specific T‐cell activation. TBP did not induce any quinone formation, probably due to its low or absent reactivity with tyrosinase, and only induced broad T‐cell activation that was not pigment cell specific. This suggests that quinone formation may contribute to the immunizing ability specifically against pigmented cells.

**Table 2:** Summary of biochemical, cellular, and immunizing effects of 4‐substituted phenols, relative to monobenzone (BOP)

Compound	Abbrev.	Ether link	Toxicity	Tyrosinase inhibition^a^	Melanin inhibition^b^	Quinone formation^c^	CD8 T‐cell activation^d^	CD4 T‐cell activation^d^	Pigment spec. T‐cell act.^e^	Pigment cell spec. CD8 T‐cell response^f^
4‐Benzyloxyphenol^g^	BOP	yes	ref^h^	ref	ref	ref	ref	ref	ref	ref
4‐Phenoxyphenol	POP	yes	−1	0	0	1	1	1	0	1
4‐Methoxyphenol	MOP	yes	−1	−1	1	0	0	0	−1	−1
4‐(n‐Hexyl)oxyphenol	HOP	yes	1	−1	−2	2	1	−1	−1	0
4‐Benzylphenol	BP	no	−1	1	0	0	0	1	0	0
4‐Phenylphenol	PhP	no	−2	1	1	−1	1	2	−1	0
4‐Methylphenol	MP	no	−2	−1	0	2	0	1	1	1
4‐(n‐Pentyl)phenol	PP	no	−1	0	1	1	0	0	−1	−1
4‐(*tert*‐Butyl)phenol	TBP	no	−2	−2	2	neg^i^	2	2	−2	0

a As measured by the MBTH assay (Figure 1).

b As measured by inhibition of cellular melanin synthesis (Figure 4).

c As measured by GSH depletion (Figure 2).

d T‐cell activation ORR (Table 1).

e Pigment cell‐specific T‐cell activation, as estimated by T‐cell reactivity against pigmented cells relative to HaCat cells (Figure 6).

f Pigment cell‐specific CD8 T‐cell activation (=CD8 T cell act +pigment spec T cell act.)

g Also known as monobenzone.

h Results are related to BOP as reference, by values −1 or −2: (much) weaker than BOP; value 0: comparable to BOP; values 1 and 2: (much)stronger than BOP.

i Neg, negative, no quinone formation.

## 4 DISCUSSION

We evaluated a series of 4‐substituted phenols for their biochemical, cellular, and immunizing ability against pigmented cells. The observed differences in these characteristics among phenols were not related to the presence or absence of an ether linkage at the fourth position. All phenols tested inhibited melanin synthesis in pigmented melanoma cells. Four phenols, all lacking an ether linkage, decreased tyrosinase protein levels in pigmented cells. Eight of ten phenols tested inhibited tyrosinase activity and induced GSH depletion by phenol‐derived reactive quinone binding, but the strength of tyrosinase inhibition did not directly correlate with the level of GSH depletion. Hydroquinone did not inhibit tyrosinase activity. No GSH depletion was found with hydroquinone or TBP. Hydroquinone has been described as oxidizable substrate for tyrosinase, rather than tyrosinase inhibitor (Passi & Nazzaro‐Porro, 1981). Depigmentation by hydroquinone is thought to result from the oxidative damage of its oxidation product 1,4‐benzoquinone to melanocytes (Briganti, Camera, & Picardo, 2003; Passi & Nazzaro‐Porro, 1981). More recent studies support our findings of the deviating behavior of hydroquinone regarding the inhibition of tyrosinase activity and GSH depletion (Ramsden & Riley, 2014; Stratford, Ramsden, & Riley, 2012). TBP has been described to interfere with melanin synthesis at the level of tyrosinase‐related protein 1 (TRP‐1), which regulates tyrosinase protein stability and its catalytic activity (Manga et al., 2006).

GSH binding of phenol‐derived quinone was tested as a marker for protein thiol binding. This can result in protein‐hapten complex formation of melanosomal proteins that have increased immunogenicity. Indeed, all phenols that induced GSH depletion also induced pigment‐specific immunity, suggesting that quinone formation contributes to the immunizing ability. The level of tyrosinase inhibition and GSH depletion varied among phenols, but this did not directly correlate with the level of immunizing ability. In addition, GSH depletion by phenols upon interaction with tyrosinase may also occur directly in vivo, thereby inducing oxidative stress in the skin. This effect likely contributes to the induction of pigment cell‐specific autoimmunity, as oxidative stress is also found in vitiligo skin and involved in vitiligo pathogenesis (Schallreuter et al., 2008).

With regard to the immunizing ability, we found that all nine phenols tested were able to induce CD8+ and/or CD4+ T cell activation against phenol‐exposed pigmented cells in a substantial proportion of healthy donors, even at low doses of IC12.5. The resulting T‐cell responses were raised against phenol‐exposed pigmented cells, but could also recognize unexposed pigmented cells. The responses were specific for pigmented cells, as less reactivity was seen against keratinocytes. This means that phenol exposure increases the immunogenicity of pigmented cells, as compared to unexposed cells, and thereby lowers the threshold for autoimmunity specifically against pigmented cells. Upon systemic spread, phenol‐induced T‐cell responses could thus induce depigmentation (and vitiligo) at distant unexposed body sites. This suggests that these phenols all confer a potential risk of skin‐bleaching and vitiligo upon skin exposure. Considering the variety in phenols we tested for their immunizing ability, this mechanism of action may also apply to other 4‐substituted phenols with known skin‐bleaching activity (Bleehen et al., 1968; Fisher, 2001).

We tested the immunizing ability of phenols at equal levels of toxicity, to exclude the known influence of cell death per se on the immunization level. However, HOP is more toxic than BOP, and our data indicate that skin exposure to HOP can already induce T‐cell activation at low doses. The other phenols were less toxic than BOP, suggesting that the risk of immunization may be most prevalent at higher doses of skin exposure or at higher exposure frequency.

TBP induces T‐cell responses against TBP‐exposed pigmented cells, which also recognized TBP‐exposed keratinocytes. T‐cell responses induced by TBP were generally more reactive against TBP‐exposed cells than unexposed cells, regardless of cell type. This indicates the reactivity was mostly directed against TBP only and corresponds to the mechanism of action of contact sensitizers that form haptens to cysteine containing proteins as carrier and raise hapten‐specific immunity (Gorbachev & Fairchild, 2001). Indeed, a patient at our institute with TBP‐induced vitiligo showed a positive delayed type hypersensitivity reaction to skin TBP exposure (Vrijman et al., 2019).

This study shows the immunizing ability of known skin‐bleaching compound MOP. This indicates that, similar to BOP, the mechanism of action of vitiligo induction or depigmentation therapy by MOP includes both melanin synthesis inhibition and immunization. Our data also indicate the immunizing ability of 4‐substituted phenols that were chosen based on the structural similarity to BOP, but were not previously associated with skin‐bleaching and are new to the pigment cell research field.

The eight phenols that induced pigment cell‐specific immunity may be applicable to induce antimelanoma immunity, similar to monobenzone (BOP) (van den Boorn et al., 2010; van den Boorn, Melief et al., 2011). In view of the risk of undesired off‐target immune activation in melanoma patients, TBP is less attractive for melanoma immunotherapy, because of the hapten‐specific response, being not restricted to pigmented cells. The other phenols tested in this study did not differ greatly in immunizing ability from monobenzone and none of the phenols was largely superior to monobenzone. This justifies the choice of monobenzone for antimelanoma immunotherapy development.

## CONFLICT OF INTEREST

Authors have no conflict of interest.

## References

[pcmr12774-bib-0001] Bleehen, S. S. , Pathak, M. A. , Hori, Y. , & Fitzpatrick, T. B. (1968). Depigmentation of skin with 4‐isopropylcatechol, mercaptoamines, and other compounds. The Journal of Investigative Dermatology, 50(2), 103–117. 10.1038/jid.1968.13 5641641

[pcmr12774-bib-0002] Boissy, R. E. , & Manga, P. (2004). On the etiology of contact/occupational vitiligo. Pigment Cell Research, 17(3), 208–214. 10.1111/j.1600-0749.2004.00130.x 15140065

[pcmr12774-bib-0003] Briganti, S. , Camera, E. , & Picardo, M. (2003). Chemical and instrumental approaches to treat hyperpigmentation. Pigment Cell Research, 16(2), 101–110. 10.1034/j.1600-0749.2003.00029.x 12622786

[pcmr12774-bib-0004] Cohn, V. H. , & Lyle, J. (1966). A fluorometric assay for glutathione. Analytical Biochemistry, 14(3), 434–440. 10.1016/0003-2697(66)90286-7 5944947

[pcmr12774-bib-0005] Cooksey, C. J. , Jimbow, K. , Land, E. J. , & Riley, P. A. (1992). Reactivity of orthoquinones involved in tyrosinase‐dependent cytotoxicity: Differences between alkylthio‐ and alkoxy‐substituents. Melanoma Research, 2(5–6), 283–293. 10.1097/00008390-199212000-00001 1337996

[pcmr12774-bib-0006] de Bruin, A. , Cornelissen, P. W. , Kirchmaier, B. C. , Mokry, M. , Iich, E. , Nirmala, E. , … Bakker, W. J. (2016). Genome‐wide analysis reveals NRP1 as a direct HIF1alpha‐E2F7 target in the regulation of motor neuron guidance in vivo. Nucleic Acids Research, 44(8), 3549–3566. 26681691 10.1093/nar/gkv1471 PMC4856960

[pcmr12774-bib-0007] de Jong, E. C. , Vieira, P. l. , Kalinski, P. , Schuitemaker, J. H. N. , Tanaka, Y. , Wierenga, E. A. , … Kapsenberg, M. l. (2002). Microbial compounds selectively induce Th1 cell‐promoting or Th2 cell‐promoting dendritic cells in vitro with diverse th cell‐polarizing signals. The Journal of Immunology, 168(4), 1704–1709. 10.4049/jimmunol.168.4.1704 11823500

[pcmr12774-bib-0008] Ewens, S. , Wulferink, M. , Goebel, C. , & Gleichmann, E. (1999). T cell‐dependent immune reactions to reactive benzene metabolites in mice. Archives of Toxicology, 73(3), 159–167. 10.1007/s002040050601 10401682

[pcmr12774-bib-0009] Fisher, A. A. (2001). In Rietschel R. L., Fowler J. F., & (Ed.), Contact Dermatitis (Fifth Edition, (Eds.), Contact leukoderma (vitiligo), hyperpigmentation and discolorations from contactants (pp. 571–579). Philadelphia, PA: Lippincott Williams & Wilkins.

[pcmr12774-bib-0010] Garcia Canovas, F. , Tudela, J. , Martinez Madrid, C. , Varon, R. , Garcia Carmona, F. , & Lozano, J. A. (1987). Kinetic study on the suicide inactivation of tyrosinase induced by catechol. Biochimica Et Biophysica Acta, 912(3), 417–423. 10.1016/0167-4838(87)90047-1 3105585

[pcmr12774-bib-0011] Gorbachev, A. V. , & Fairchild, R. L. (2001). Induction and regulation of T‐cell priming for contact hypersensitivity. Critical Reviews in Immunology, 21(5), 451–472. 10.1615/CritRevImmunol.v21.i5.30 11942559

[pcmr12774-bib-0012] Halaban, R. , Cheng, E. , Svedine, S. , Aron, R. , & Hebert, D. N. (2001). Proper folding and endoplasmic reticulum to golgi transport of tyrosinase are induced by its substrates, DOPA and tyrosine. Journal of Biological Chemistry, 276, 11933–11938. 11124258 10.1074/jbc.M008703200

[pcmr12774-bib-0013] Hariharan, V. , Klarquist, J. , Reust, M. J. , Koshoffer, A. , McKee, M. D. , Boissy, R. E. , & Le Poole, I. C. (2010). Monobenzyl ether of hydroquinone and 4‐tertiary butyl phenol activate markedly different physiological responses in melanocytes: Relevance to skin depigmentation. The Journal of Investigative Dermatology, 130(1), 211–220. 10.1038/jid.2009.214 19657355

[pcmr12774-bib-0014] Kroll, T. M. , Bommiasamy, H. , Boissy, R. E. , Hernandez, C. , Nickoloff, B. J. , Mestril, R. , & Caroline Le Poole, I. (2005). 4‐Tertiary butyl phenol exposure sensitizes human melanocytes to dendritic cell‐mediated killing: Relevance to vitiligo. The Journal of Investigative Dermatology, 124(4), 798–806. 10.1111/j.0022-202X.2005.23653.x 15816839 PMC1747533

[pcmr12774-bib-0015] Manga, P. , Sheyn, D. , Yang, F. , Sarangarajan, R. , & Boissy, R. E. (2006). A role for tyrosinase‐related protein 1 in 4‐tert‐butylphenol‐induced toxicity in melanocytes: Implications for vitiligo. American Journal of Pathology, 169(5), 1652–1662. 10.2353/ajpath.2006.050769 17071589 PMC1780195

[pcmr12774-bib-0016] Manini, P. , Napolitano, A. , Westerhof, W. , Riley, P. A. , & d'Ischia, M. (2009). A reactive ortho‐quinone generated by tyrosinase‐catalyzed oxidation of the skin depigmenting agent monobenzone: Self‐coupling and thiol‐conjugation reactions and possible implications for melanocyte toxicity. Chemical Research in Toxicology, 22(8), 1398–1405. 19610592 10.1021/tx900018q

[pcmr12774-bib-0017] Menter, J. M. , Etemadi, A. A. , Chapman, W. , Hollins, T. D. , & Willis, I. (1993). In vivo depigmentation by hydroxybenzene derivatives. Melanoma Research, 3(6), 443–449. 10.1097/00008390-199311000-00007 8161883

[pcmr12774-bib-0018] Naish, S. , Cooksey, C. J. , & Riley, P. A. (1988). Initial mushroom tyrosinase‐catalysed oxidation product of 4‐hydroxyanisole is 4‐methoxy‐ortho‐benzoquinone. Pigment Cell Research, 1(6), 379–381. 3148921 10.1111/j.1600-0749.1988.tb00138.x

[pcmr12774-bib-0019] Naish, S. , Holden, J. L. , Cooksey, C. J. , & Riley, P. A. (1988). Major primary cytotoxic product of 4‐hydroxyanisole oxidation by mushroom tyrosinase is 4‐methoxy ortho benzoquinone. Pigment Cell Research, 1(6), 382–385. 3148922 10.1111/j.1600-0749.1988.tb00139.x

[pcmr12774-bib-0020] Nazih, A. , Benezra, C. , & Lepoittevin, J. P. (1993). Bihaptens with 5‐ and 6‐methyl‐substituted alkylcatechols and methylene lactone functional groups: Tools for hapten (allergen or tolerogen)‐protein interaction studies. Chemical Research in Toxicology, 6(2), 215–222. 10.1021/tx00032a011 8477012

[pcmr12774-bib-0021] Njoo, M. D. , Vodegel, R. M. , & Westerhof, W. (2000). Depigmentation therapy in vitiligo universalis with topical 4‐methoxyphenol and the Q‐switched ruby laser. Journal of the American Academy of Dermatology, 42(5 Pt 1), 760–769. 10.1067/mjd.2000.103813 10775851

[pcmr12774-bib-0022] Passi, S. , & Nazzaro‐Porro, M. (1981). Molecular basis of substrate and inhibitory specificity of tyrosinase: Phenolic compounds. British Journal of Dermatology, 104(6), 659–665. 10.1111/j.1365-2133.1981.tb00752.x 6788064

[pcmr12774-bib-0023] Ramsden, C. A. , & Riley, P. A. (2014). Mechanistic aspects of the tyrosinase oxidation of hydroquinone. Bioorganic & Medicinal Chemistry Letters, 24(11), 2463–2464. 10.1016/j.bmcl.2014.04.009 24767847

[pcmr12774-bib-0024] Schallreuter, K. U. , Bahadoran, P. , Picardo, M. , Slominski, A. , Elassiuty, Y. E. , Kemp, E. H. , … Westerhof, W. (2008). Vitiligo pathogenesis: Autoimmune disease, genetic defect, excessive reactive oxygen species, calcium imbalance, or what else? Experimental Dermatology, 17(2), 139–140. 18205713 10.1111/j.1600-0625.2007.00666_1.x

[pcmr12774-bib-0025] Smit, N. P. , Peters, K. , Menko, W. , Westerhof, W. , Pavel, S. , & Riley, P. A. (1992). Cytotoxicity of a selected series of substituted phenols towards cultured melanoma cells. Melanoma Research, 2(5–6), 295–304. 10.1097/00008390-199212000-00002 1292781

[pcmr12774-bib-0026] Stratford, M. R. , Ramsden, C. A. , & Riley, P. A. (2012). The influence of hydroquinone on tyrosinase kinetics. Bioorganic & Medicinal Chemistry, 20(14), 4364–4370. 10.1016/j.bmc.2012.05.041 22698780

[pcmr12774-bib-0027] Tayama, K. , & Takahama, M. (2002). Depigmenting action of phenylhydroquinone, an O‐phenylphenol metabolite, on the skin of JY‐4 black guinea‐pigs. Pigment Cell Research, 15(6), 447–453. 10.1034/j.1600-0749.2002.02057.x 12453187

[pcmr12774-bib-0028] Teulings, H.‐E. , Tjin, E. P. M. , Willemsen, K. J. , van der Kleij, S. , Meulen, S. T. , Kemp, E. H. , … Luiten, R. M. (2018). Anti‐Melanoma immunity and local regression of cutaneous metastases in melanoma patients treated with monobenzone and imiquimod; a phase 2 a trial. OncoImmunology, 7(4), e1419113 10.1080/2162402X.2017.1419113 29632737 PMC5889200

[pcmr12774-bib-0029] Tjin, E. p. m. , Konijnenberg, D. , Krebbers, G. , Mallo, H. , Drijfhout, J. w. , Franken, K. l. m. c. , … Luiten, R. m. (2011). T‐cell immune function in tumor, skin, and peripheral blood of advanced stage melanoma patients: Implications for immunotherapy. Clinical Cancer Research, 17(17), 5736–5747. 10.1158/1078-0432.CCR-11-0230 21750202

[pcmr12774-bib-0030] van den Boorn, J. G. , Konijnenberg, D. , Tjin, E. P. M. , Picavet, D. I. , Meeuwenoord, N. J. , Filippov, D. V. , … Luiten, R. M. (2010). Effective melanoma immunotherapy in mice by the skin‐depigmenting agent monobenzone and the adjuvants imiquimod and CpG. PLoS ONE, 5(5), e10626 10.1371/journal.pone.0010626 20498710 PMC2869359

[pcmr12774-bib-0031] van den Boorn, J. G. , Melief, C. J. , & Luiten, R. M. (2011). Monobenzone‐induced depigmentation: From enzymatic blockade to autoimmunity. Pigment Cell Melanoma Res, 24(4), 673–679. 21689385 10.1111/j.1755-148X.2011.00878.x

[pcmr12774-bib-0032] van den Boorn, J. G. , Picavet, D. I. , Van Swieten, P. F. , van Veen, H. A. , Konijnenberg, D. , van Veelen, P. A. , … Luiten, R. M. (2011). Skin‐Depigmenting Agent Monobenzone Induces Potent T‐Cell Autoimmunity toward Pigmented Cells by Tyrosinase Haptenation and Melanosome Autophagy. The Journal of Investigative Dermatology, 131(6), 1240–1251. 21326294 10.1038/jid.2011.16

[pcmr12774-bib-0033] Vrijman, C. , Hosseinpour, D. , Bakker, J. G. , Wolkerstorfer, A. , Bos, J. D. , van der Veen, J. P. , & Luiten, R. M. (2013). Provoking factors, including chemicals, in Dutch patients with vitiligo. British Journal of Dermatology, 168(5), 1003–1011. 10.1111/bjd.12162 23252956

[pcmr12774-bib-0034] Vrijman, C. , Willemsen, K. J. , Tjin, E. P. M. , Kammeyer, A. , van den Boorn, J. G. , van der Veen, J. P. W. … Luiten, RM (2019). T‐cell responses against 4‐tertiarybutylphenol‐exposed pigmented cells in a patient with occupational vitiligo. British Journal of Dermatology, https://doi.org/ 10.1111/bjd.17713. [Epub ahead of print] 10.1111/bjd.17713 PMC6850457 30719700

[pcmr12774-bib-0035] Webb, K. C. , Eby, J. M. , Hariharan, V. , Hernandez, C. , Luiten, R. M. , & Le Poole, I. C. (2014). Enhanced bleaching treatment: Opportunities for immune‐assisted melanocyte suicide in vitiligo. Experimental Dermatology, 23(8), 529–533. 10.1111/exd.12449 24840876 PMC4126600

[pcmr12774-bib-0036] Winder, A. J. , & Harris, H. (1991). New assays for the tyrosine hydroxylase and dopa oxidase activities of tyrosinase. European Journal of Biochemistry, 198(2), 317–326. 10.1111/j.1432-1033.1991.tb16018.x 1674912

[pcmr12774-bib-0037] Yang, F. , & Boissy, R. E. (1999). Effects of 4‐tertiary butylphenol on the tyrosinase activity in human melanocytes. Pigment Cell Research, 12(4), 237–245. 10.1111/j.1600-0749.1999.tb00756.x 10454291

[pcmr12774-bib-0038] Yang, F. , Sarangarajan, R. , Le Poole , I. C. , Medrano, E. E. , & Boissy, R. E. (2000). The cytotoxicity and apoptosis induced by 4‐tertiary butylphenol in human melanocytes are independent of tyrosinase activity. The Journal of Investigative Dermatology, 114(1), 157–164. 10.1046/j.1523-1747.2000.00836.x 10620132

