# Supplementary figures and images for: Mechanism of action of 4‐substituted phenols to induce vitiligo and antimelanoma immunity
Source: Pigment Cell Melanoma Res. 2019 Mar 18;32(4):540–52. doi: 10.1111/pcmr.12774 (PMC6850206; doi:10.1111/pcmr.12774)

**A****PP**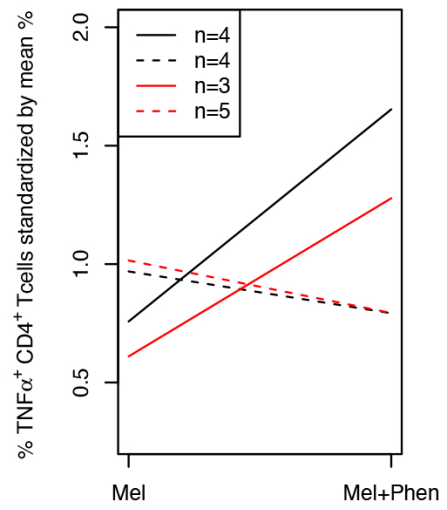**PhP**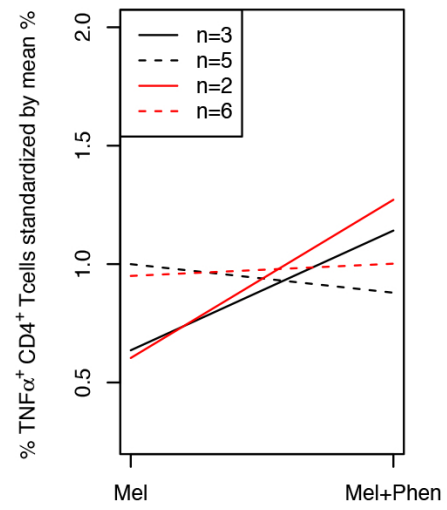**POP**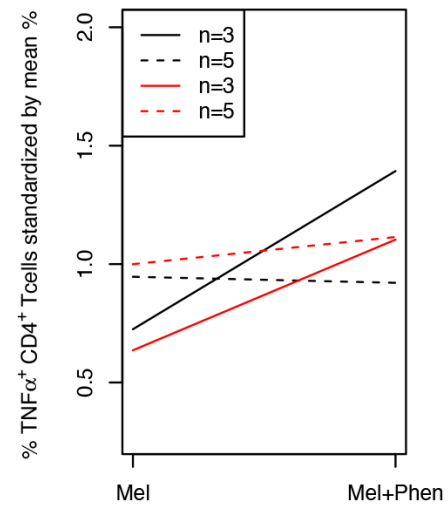**BP**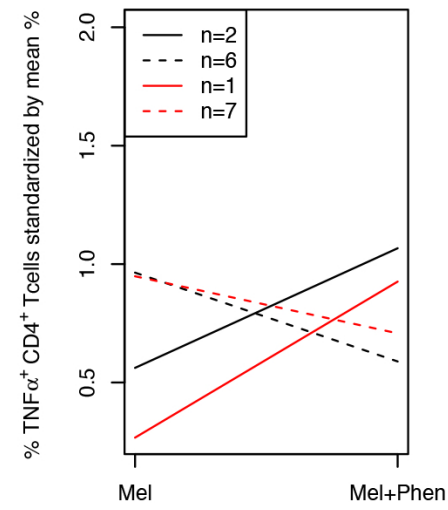**BOP**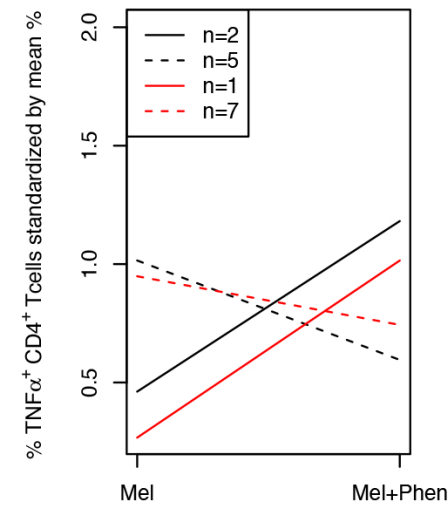**TBP**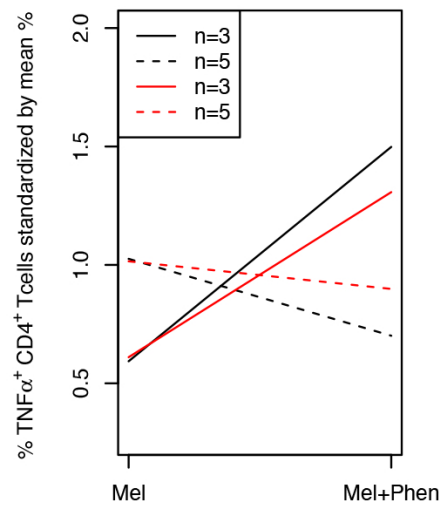**MP**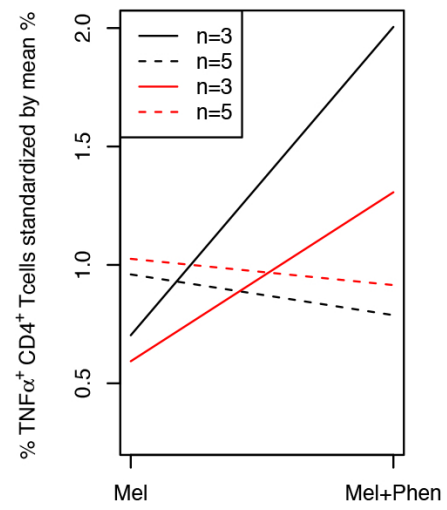**MOP**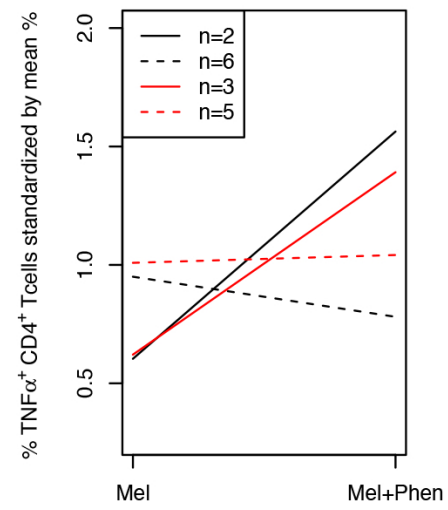**HOP**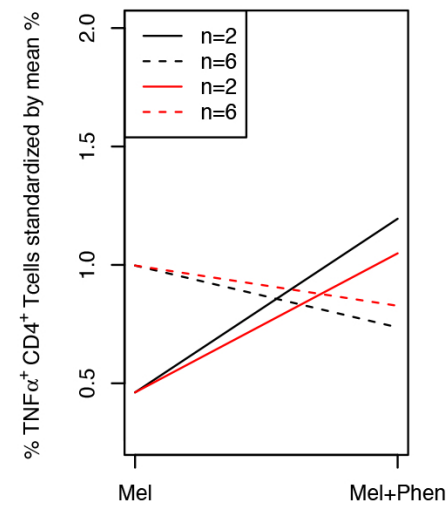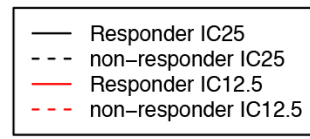

**B****PP**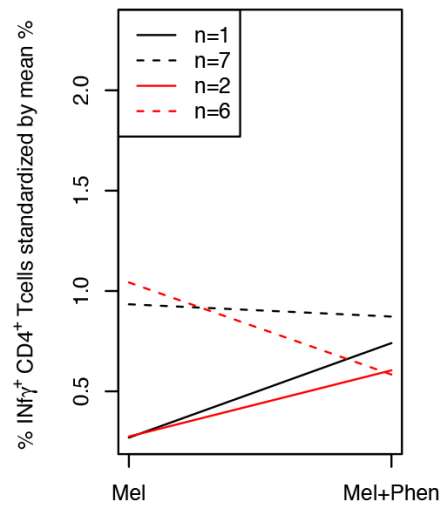**PhP**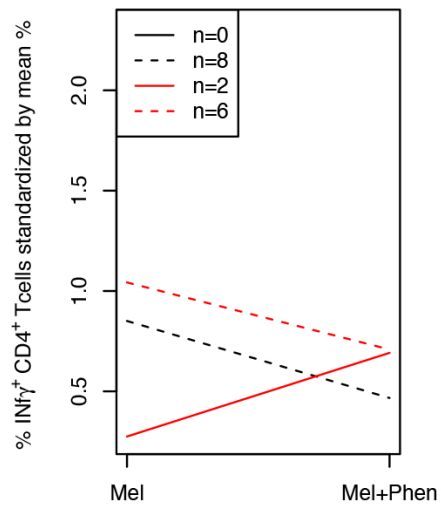**POP**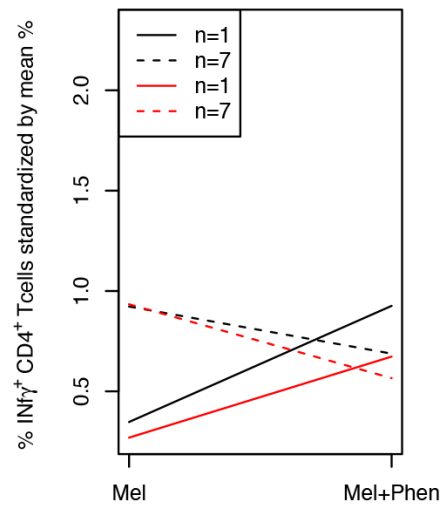**BP**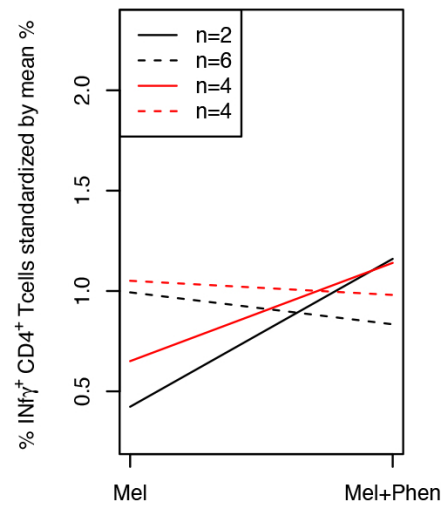**BOP**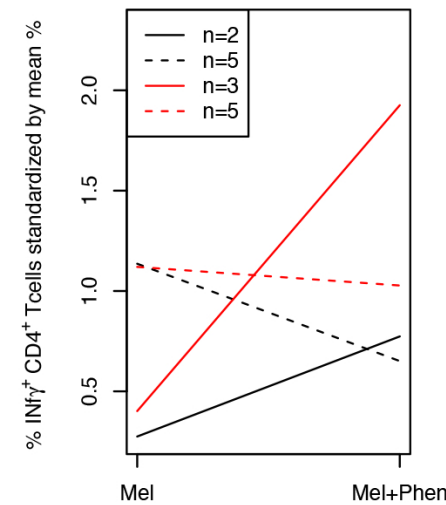**TBP**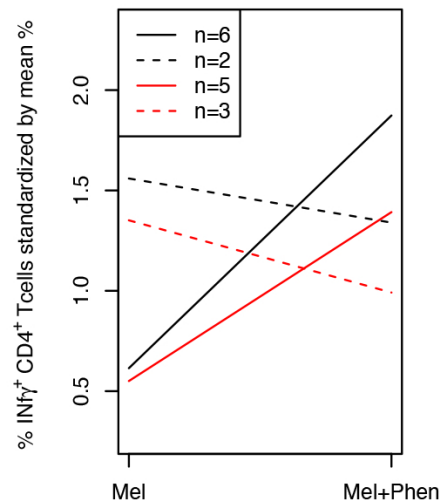**MP**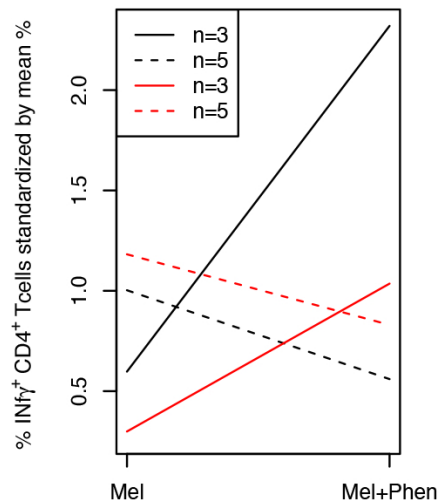**MOP**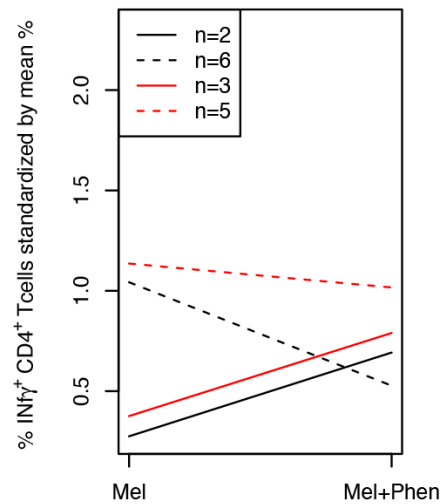**HOP**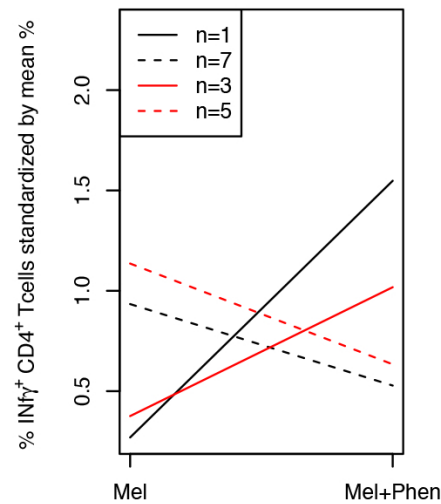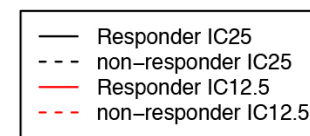

**C**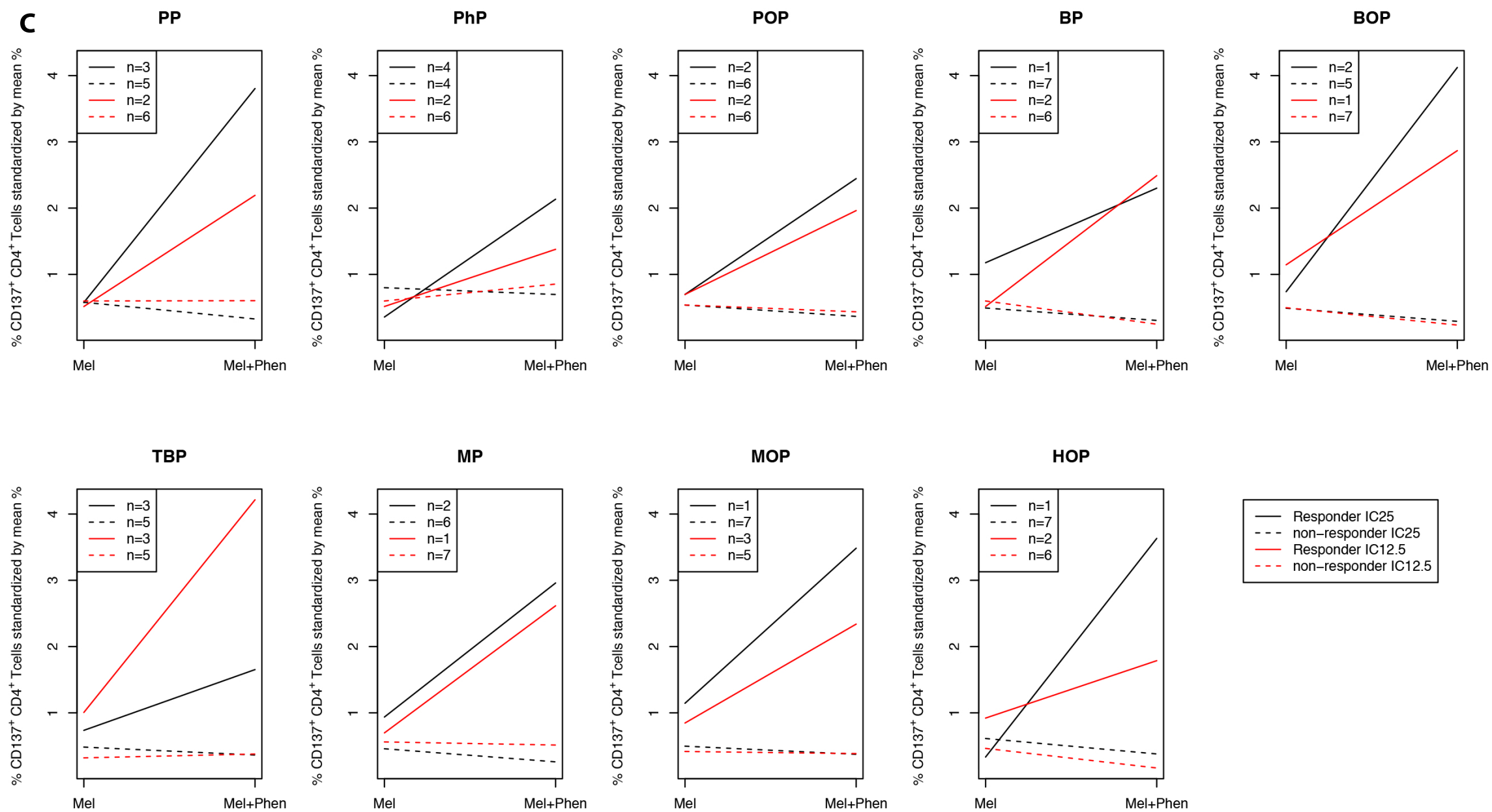

Supplement: Supplementary file 1 [file PCMR-32-540-s001.pdf]
